# Post-laser in situ keratomileusis interface *Arthrographis kalrae* keratitis

**DOI:** 10.5935/0004-2749.20200004

**Published:** 2020

**Authors:** Yolanda Fernández-Barrientos, Antonio Ramos-Suárez, Fernando Fernández-Sánchez, Antonio Tirado-Carmona

**Affiliations:** 1 Ophthalmology Service, Health Agency Costa del Sol, Marbella, Málaga, Spain; 2 Microbiology Service, Health Agency Costa del Sol, Marbella, Málaga, Spain; 3 Ophthalmology Institute Costa del Sol, Fuengirola, Málaga, Spain

**Keywords:** Keratitis/microbiology, Eye infections, fungal, Keratomileusis, laser in situ, Refractive surgical procedures, Antifungal agents, Postoperative complications, Ceratite/microbiologia, Infecções oculares fúngicas, Ceratomileuse assistida por excimer laser in situ, Procedimen tos cirúrgicos refrativos, Antifúngicos, Complicações pós-operatórias

## Abstract

We describe a case of keratomycosis caused by *Arthographis kalrae* after excimer laser keratomileusis. A 38-year-old female developed stromal keratitis eight weeks after refractive surgery. She developed severe corneal stromal infiltration and mild anterior segment inflammation, which could not be treated with topical voriconazole 1%, but topical natamycin 5% ameliorated her condition. A reactivation of keratomycosis symptoms was observed; therefore, longer treatment was administered to the patient. It has been reported that *A. kalrae* keratomycosis is associated with exposure to soil and contact lens usage. However, the patient, who lived in a rural location, was neither involved in gardening activities nor had a history of wearing contact lenses. This is the first case of post-refractive *A. kalrae* keratomycosis.

## Abstract (pt)

Descrevemos um caso de ceratomicose por *Arthographis kalrae* após ceratomileusis por excimer laser. Uma mulher de 38 anos desenvolveu ceratite estromal oito semanas após a cirurgia refrativa. Ela desenvolveu infiltração estromal grave da córnea e uma leve inflamação do segmento anterior, que não pode ser tratada com voriconazol tópico a 1%, mas a natamicina tópica a 5% melhorou sua condição. Uma reativação dos síntomas de ceratomicose foi observada; portanto, tratamento mais prolongado foi administrado a paciente. Tem sido relatado que a ceratomicose por A. *kalrae* está associada à exposição ao solo e ao uso de lentes de contato. No entanto, a paciente, que vivía em um local rural, não estava envolvida em atividades de jardinagem e nem tinha histórico de uso de lentes de contato. Este é o primeiro caso de ceratomicose pós-refrativa por *A. kalrae*.

## INTRODUCTION

Fungal keratitis often manifests into an indolent infection, and usually patients are given inappropriate treatment. *Arthrographis kalrae* is a filamentous fungus isolated from soil and compost. It has rarely been reported as an opportunistic pathogen in humans. It is difficult to perform microbial differentiation because of its dimorphism^(1)^. We describe the first case of *A. kalrae* keratomycosis after excimer laser keratomileusis (LASIK).

## CASE REPORT

**Figure 1:**
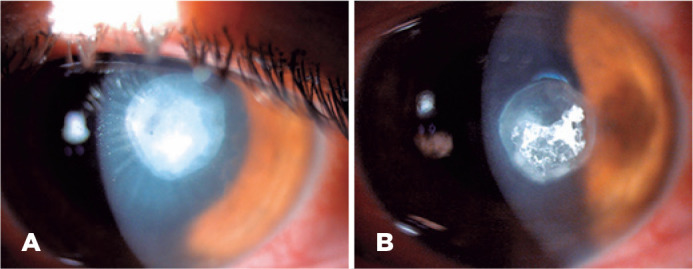
A) The patient manifested a best corrected visual acuity of 20/60, corneal epithelial defect with dense stromal infiltrate situated paracentral affecting without visual axis, and stromal folds with 1+ Tyndall effect. B) After one week of treatment with topical natamycin 5% and systemic doxycycline 100 mg/day, the infiltration started to improve.

**Figure 2:**
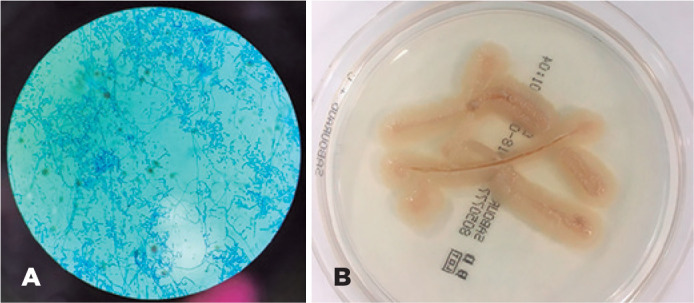
A) Microscopic morphology of *A. kalrae* (Lactopheno Color Blue mount shows lateral short cell of hyaline hyphae and hyaline smooth-walled arthroconidia at 100× magnification). B) *A. kalrae* appears as Cream-like colony with fine, velvety appearance after incubation at 37°C on Saboureaud agar supplemented with chloramphenicol for 4 days .

A 38-year-old healthy immunocompetent woman was referred to our hospital for persistent corneal ulcer after two weeks of topical treatment with ceftazidime 50 mg/ml and tobramycin 14 mg/ml. She had undergone bilateral LASIK procedure two months before her symptoms developed. The patient complained of decreased vision, ocular pain, and photophobia. She declared no history of gardening activities or soil exposure; the only risk factor was living in a rural area. On presentation, her best corrected visual acuity (BCVA) was 20/60. Additionally, corneal epithelial defect with dense stromal infiltrate of 1 mm diameter situated paracentral without affecting visual axis, stromal folds, and 1+ Tyndall effect were observed (Figure 1 A). A corneal culture test was conducted, and topical and systemic voriconazole (topical concentration of 1% and oral dose of 400 mg/day) was administered to the patient. *A. kalrae* was identified by assessing the colony and microscopic morphologies of cornea scrapping cultures after three days of growth on Mycosel agar, potato glucose agar, and Saboureaud agar supplemented with chloramphenicol (Figure 2). The second corneal scraping was subjected to matrix-assisted laser desorption ionization-time of flight (MALDI-TOF) mass spectrometry (Bruker Daltonics MALDI Biotyper; Billerica, MA - USA), and the presence of *A kalrae* was confirmed. The spectra were analyzed by the MALDI Biotyper software version 1.0.3.0 (Bruker Daltonics) according to the method described by Cassagne et al.^(2)^. Two weeks after the treatment, the size of the ulcer increased to 3 mm in diameter and the presence of anterior corneal melting required a corneal debridement. Topical natamycin 5% every hour and systemic doxycycline 100 mg/day were then added to the treatment. After one week of treatment with natamycin 5%, the stromal infiltration improved (Figure 1 B), and medication dosage was reduced for 4 weeks. At that time, an irregular corneal leucoma developed, and BCVA was 20/30.

Two months following the treatment, she again complained of decreased vision and ocular discomfort. The BCVA was 20/60, and dense stromal corneal infiltration with 2+ Tyndall effect in the anterior chamber was observed. Hence, topical treatment with natamycin 5% every hour and systemic voriconazole 400 mg/day were administered to the patient. Two weeks after the treatment, BCVA improved to 20/30. Further, a residual leucoma in the pericentral cornea with no inflammation in the anterior chamber was observed. Thereafter, the topical natamycin 5% treatment was reduced for eight weeks and the patient was followed-up regularly.

## DISCUSSION

*Arthrographis* is a genus linked to *Malbranchea*. The characteristic of the species is the presence of one-celled, hyaline, smooth-walled, and cylindrical arthroconidia directly formed by fragmentation of undifferentiated hyphae or for the fresh cultures by disjunction and segmentation of hyaline fertile branches borne at the apex of the conidiophore. Mature arthroconidia become bigger and elongated. In addition, single-celled, hyaline, smooth, and spherical blastoconidia occur directly on the sides of undifferentiated hyphae or on short pedicels. The genus *Arthrographis* consists of five species: *A. kalrae, A. cuboidea, A. lignicola, A. pinicola*, and *A. alba. A. kalrae* is a saprophytic fungus and it is distributed worldwide. Its identification is difficult by microscopy alone. *A. kalrae* rarely causes human diseases; only four cases of ocular infection have been reported thus far (Table 1). Risk factors of ocular infection include ocular trauma by foreign body inoculation, wearing of contact lens, and diabetes. None of the previous cases have described refractive surgery. Recently, some cases of opportunistic infections caused by *A. kalrae* have been described (Table 2)^(3^-^5)^.

**Table 1:** Description of the reported cases of *A. kalrae* infection.

Case Report	Risk factors	Visual acuity	Clinical findings	Topical and systemic antibiotics
Perlman et al.^(1)^, 1997.	Soft contact lens Gardening activities	Initial 20/20 Final 20/20	Epithelial defect corneal Stromal haze Corneal infiltrate Not feathery margins Endotelial plaque Anterior chamber reaction	Topical anphotericin B 0.15% Oral Ketoconazole 100 mg/24h
Biser et al.^(3)^, 2004.	Dailies toric contact lens Gardening Rinitis treated with oral loratadina and fluticasona nasal spray	Initial 20/50 Final 20/200	Initial conjuntivitis Stromal haze and corneal edema	Topical anphotericin B 0.4% Miconazol 10% Itraconazol 200 mg/12h
Thomas et al.^(4)^, 2011.	Soft contacts lens	Initial 20/50 Final 20/100	Epithelial defect Corneal infiltrate Corneal edema Endothelial precipitates Anterior chamber reaction	Topical voriconazole 1 % Oral Voriconazole 200 mg/12h Intravenous voriconazole >2 µg/mL Intracameral voriconazole 4QPP
Ramli et al.^(5)^, 2013.	Diabetes Accidental foreign body inoculation.	Initial 20/40 Final Perception to light	Corneal perforation Epitelial infiltrate, feathery stromal infitratation, surrounding edema and endothelial plaque Bad evolution, perforation	Oral Vibracina 100 mg/day + oral fluconazol 200 mg/day Topical amphotericin B + topical fluconazole Topical QPP
Fernandez-Barrientos et al^(9)^, 2019.	Rural village resident Late postoperative corneal refractive surgery	Initial 20/80 Final 20/80	Estromal infiltrate Meelting flap over inflitated Anterior chamber reaction	Topical natamycin 5% topical Oral voriconazole 200 mg/12h oral

**Table 2:** Cases of opportunistic infections caused by *A. Kalrae*.

Case report	Site of infection	Treatment
Degarve et al^(10)^, 1997.	Mycetoma	Itraconazole (4 months), resolved.
Chin-Hong, et al^(11)^, 2001.	Sinusitis and meningitis in AIDS	Itraconazole Death after resolved
Xi et al<^12)^, 2004.	Ethmoid sinusitis and ophthalmitis	Surgical + intravenous amphotericin B 50 mg/day, nystatin and topical fluconazol 0.2%; itraconazole (4 weeks); relapse; surgical + itraconazole 400 mg/day (3 weeks) Resolved, evisceration was required.
Pichon et al^(13)^, 2008.	Cerebral vasculitis and meningitis	None Postmorten diagnostic
Volleková et al^(14)^, 2008.	Onychomycosis	
Sugiura et al^(15)^, 2010.	Onychomycosis	Oral terbinafine 125 mg/day + miconazole 1% topical (7 months), resolved
Diego Candela et al^(16)^, 2010.	Endocarditis	Surgical + amphotericin B, voriconazole, posaconazole (4 months); relapse; posaconazol (6 months); relapse embolic phenomena and death
Vos et al^(17)^, 2012.	Lung infection, patient history of lymphoma	Lobectomy + intraconazol (2 weeks), resolved
Boan et al^(18)^, 2012.	Native knee joint infection	Amphotericin B, posaconazole, terbinafine Resolved after 18 months
Shainaghi et al^(19)^, 2015.	Knee arthitis after penetrating wound	Voriconazole, resolved
Ong et al^(20)^, 2014.	Knee arthitis after penetrating injury	Protesis, resolved
Denis et al^(21)^, 2016.	Fungemia after lung transplantation	Capsofucina 50 mg/day, intravenous amphotericin B 3 mg/kg/day, death
Campoverde-Espinoza J et al^(22)^, 2017.		Posaconazole, resolved

Due to the intense stromal infiltration and mixed characteristics, the initial clinical diagnosis of mycotic keratitis was superficial and difficult. The final diagnosis of *A. kalrae* keratitis was made after the identification of *A. kalrae* by colony and microscopic morphologies of the cornea scrapping cultures, and confirmation of *A. kalrae*identity by MALDI-TOF.

No data were available regarding the most appropriate treatment for *A. kalrae* infection. Previous *in vitro* studies on the antifungal susceptibility of clinical isolates of *A. kalrae* show that terbinafine is highly active against *A. kalrae*, followed by azoles (particularly posaconazole); additionally, amphotericin B exerted low antifungal activity, whereas echinocandins showed almost no antifungal activity^(6)^. *In vivo* studies showed different results with voriconazole 1% and natamycin 5%. As the infection in this case as severe and the patient did not respond to voriconazole 1%, topical natamycin 5% was administered to the patient and a positive response was observed.

In this case, flap lift during the course of infection treatment could have improved the efficacy of the antibiotic and thereby ameliorated *A. kalrae* keratitis. A microbiology-based treatment, such as interface scraping after a flap lift, is recommended for ocular infections ^(7^,^8)^.

*A. kalrae* is a dimorphic fungus for which microbial differentiation can be difficult using common methods. Here, we report a case of post-refractive *A. kalrae* infection with susceptibility to topical natamycin 5%, which is different from what is reported in literature. To our knowledge, this is the first case report of post-refractive *A. kalrae* infection in Spain. The antifungal susceptibility of *A. kalrae* is different from what is known so far; therefore, careful treatment with regular follow-ups must be conducted.
